# Fucoidan Oligosaccharides from *Kjellmaniella crassifolia* Ameliorate Ulcerative Colitis by Regulating the TLR4 and NF-κB Signaling Pathway and Modulating Gut Microbiota

**DOI:** 10.3390/md24050186

**Published:** 2026-05-21

**Authors:** Zhiying Xu, Zheyu Jia, Liu Li, Feiyan Zeng, Jiyan Sun, Yichao Ma, Wenzheng Shi, Shu Liu, Yunhai He, Qiukuan Wang, Dandan Ren

**Affiliations:** 1 College of Food Science and Engineering, Dalian Ocean University, Dalian 116023, China; 2 National R & D Branch Center for Seaweed Processing, Dalian 116023, China; 3 Key Laboratory of Aquatic Product Processing and Utilization of Liaoning Province, Dalian 116023, China; 4 College of Food Sciences & Technology, Shanghai Ocean University, Shanghai 201306, China

**Keywords:** fucoidan, fucoidan oligosaccharides, ulcerative colitis, anti-inflammatory mechanism, gut microbiota

## Abstract

Ulcerative colitis (UC) is a form of inflammatory bowel disease (IBD), which is marked by severe abdominal pain, weight loss, perianal bleeding, and diarrhea. This study successfully isolated and purified four low-molecular-weight fucoidan oligosaccharides through acid hydrolysis and Bio Gel P10 gel filtration. The molecular weights were 2.9 × 10^4^–1.36 × 10^5^ Da, 182–1012 Da, 161–939 Da and 161–939 Da, respectively. A mouse model of colitis was induced using Dextran Sulfate Sodium (DSS). The results indicated that fucoidan and fucoidan oligosaccharides could ameliorate murine ulcerative colitis, with the oligosaccharides (200 mg/kg/d) demonstrating superior therapeutic effects. This superiority was likely attributed to the lower molecular weight and higher content of total sugars and fucose. The primary mechanisms involved the modulation of gene and protein expression levels associated with the Toll-like receptor 4, Myeloid differentiation primary response 88, nuclear factor kappa-light-chain-enhancer of activated B cells, p65, and Inhibitor of kappa light polypeptide gene enhancer in B cells, alpha (TLR4, MYD88, NF-κB p65, and IκB-α) signaling pathways, which reduce the production of inflammatory cytokines such as tumor necrosis factor-alpha, Interleukin-1 beta and Interleukin-6 (TNF-α, IL-1β, and IL-6). Additionally, these oligosaccharides alleviated oxidative stress, enhanced the levels of intestinal barrier proteins (Claudin family member 4 and Zonula occludens protein 1), regulated the abundance and diversity of the gut microbiota, and increased the levels of short-chain fatty acids (SCFAs) in the intestine. It is worth emphasizing that this study can only demonstrate that fucoidan oligosaccharides have a mitigating effect on intestinal inflammation in mice. Further research is needed in the future to investigate the structure–activity relationship of fucoidan oligosaccharides and their impact on human intestinal microbiota, in order to further elucidate their anti-inflammatory mechanisms.

## 1. Introduction

Inflammatory bowel disease (IBD) is a common gastrointestinal disorder characterized by high levels of pro-inflammatory cytokines and epithelial barrier dysfunction. IBD is a chronic inflammatory disease with no known cure [1]. The etiology of IBD is generally attributed to genetic factors that may affect the relevant genes and lead to a dysregulated immune system to the gut microbiota. IBD is subsequently triggered by environmental factors stimulating and causing mucosal barrier dysfunction. A small percentage of IBD patients develop uveitis and scleritis, with severe cases exhibiting symptoms such as blurred vision, eye pain, photophobia, and headache [2]. Arthritis occurs in 3–5% of patients, severe oral ulcers occur in 4–10% of patients, erythema nodosum occurs in 3–15% of patients, and pyoderma gangrenosum occurs in 1–12% of patients [3]. In total, 30% of IBD patients develop liver dysfunction that leads to serious diseases such as primary sclerosing cholangitis (PSC), cholangiocarcinoma, autoimmune hepatitis, IgG4 cholangitis, and idiopathic pancreatitis [4]. Nowadays, the prevalence and incidence of IBD are rapidly accelerating worldwide. Ulcerative colitis (UC) is the most common form. UC is characterized by continuous and diffuse inflammation of the colon, with ulcers of varying sizes or linear ulcers distributed throughout the intestine. Inflammation begins in the rectum and extends retrogradely towards the cecum. Chronic structural changes are present, which include crypt distortion, chronic inflammatory cell infiltration in the lamina propria and superficial submucosa, basal lymphoplasmacytosis, and Paneth cell or pyloric gland metaplasia [5]. Patients with UC have reduced gut microbiota abundance [6] and impaired barrier function of colonic epithelial cells [7,8]. Common symptoms include recurrent abdominal pain, weight loss, pathological mucosal damage and ulcers in the intestine, colon shortening, diarrhea, and bloody stools [9]. Current medical treatments include anti-inflammatory drugs such as 5-aminosalicylic acid [10], as well as immunosuppressants, biologics, and antibiotics [5,11,12,13]. Drug therapy for UC can produce certain side effects like arthritis and diabetes. Natural anti-inflammatory substances are needed for improvement with fewer side effects.

Fucoidan is mainly derived from brown algae, echinoderms, and mollusks [14]. Fucoidan is a sulfated heteropolysaccharide and primarily composed of monosaccharides such as fucose, galactose, xylose, and glucose [15]. The backbone of fucoidan can be classified as α-(1,3)-L-fucose, α-(1,3)/(1,4)-L-fucose, or sulfated galactose [16]. Fucoidan exhibits various bioactivities, which include antioxidant, anticoagulant, antitumor, and anti-inflammatory properties [17,18,19,20]. The large molecular weight, complex structure, and high viscosity of fucoidan affect the bioavailability and solubility [21]. These cannot be broken down by endogenous enzymes in the human small intestine. Fucoidan oligosaccharides improve bioavailability and exhibit stronger biological activity than fucoidan, due to the lower molecular weight [22]. The methods for preparing fucoidan oligosaccharides include acid degradation [23], ultrasonic degradation, radiation degradation [24] and enzymatic degradation [25,26].

Fucoidan and fucoidan oligosaccharides both exhibit anti-inflammatory effects. The primary mechanisms include blocking lymphocyte invasion, inhibiting various enzymes, inducing apoptosis, influencing the intestinal ecosystem, regulating the colonic microbiota, and downregulating pro-inflammatory cytokine production via signaling pathways [27,28,29]. Ekaterina D. Obluchinskaya et al. found that the anti-inflammatory mechanism of fucoidan is related to its ability to inhibit protein denaturation, and this effect is closely associated with the fucose and sulfate contents [30]. Hidenori Takahashi et al. discovered that fucoidan exerts anti-inflammatory effects in patients with advanced cancer by reducing pro-inflammatory factors such as IL-1 and IL-6 [31]. Hyo-Geun Lee et al. reported that fucoidan extracted from Padina arborescens possesses anti-inflammatory activity by suppressing the production of pro-inflammatory factors through the regulation of the inducible nitric oxide synthase/cyclooxygenase-2, mitogen-activated protein kinase and nuclear factor kappa-light-chain-enhancer of activated B cell (iNOS/COX-2, MAPK and NF-κB) signaling pathways [32]. Due to the large molecular weight, fucoidan’s passage across the cell membrane and subsequent absorption are often hindered. Therefore, fucoidan oligosaccharides obtained through the degradation of fucoidan have a smaller molecular weight, a higher fucose content, and consequently exhibit better absorption [33]. Arachchige Maheshika Kumari Jayasinghe et al. found that low-molecular-weight fucoidan exerted anti-inflammatory effects by inhibiting the NF-κB and MAPK signaling pathways [34]. Young-In Kim et al. demonstrated that low-molecular-weight fucoidan could reduce the release of IL-1 and increase IL-10 in an inflammatory model, thereby reducing oxidative stress [35].

Because fucoidan has a high molecular weight, poor solubility and low absorption efficiency, we hypothesize that fucoidan oligosaccharides—being of lower molecular weight and enriched in fucose—will exhibit stronger anti-inflammatory activity. In this study, SPF1, SPF2, SPF3, and SPF4 were isolated and purified through acid hydrolysis and Bio-Gel P10 gel column chromatography. A chemical composition analysis was conducted on fucoidan (F) and the fucoidan oligosaccharides (SPF1, SPF2, SPF3, and SPF4). Based on the results, a fucoidan oligosaccharide with specific structural characteristics was selected for subsequent animal experiments. This study aims to investigate the ameliorative effects and underlying mechanisms of fucoidan and fucoidan oligosaccharides on DSS-induced colitis, thereby providing a theoretical basis for the application of fucoidan oligosaccharides.

## 2. Results and Analysis

### 2.1. Preparation of Fucoidan Oligosaccharides

**Figure 1:**
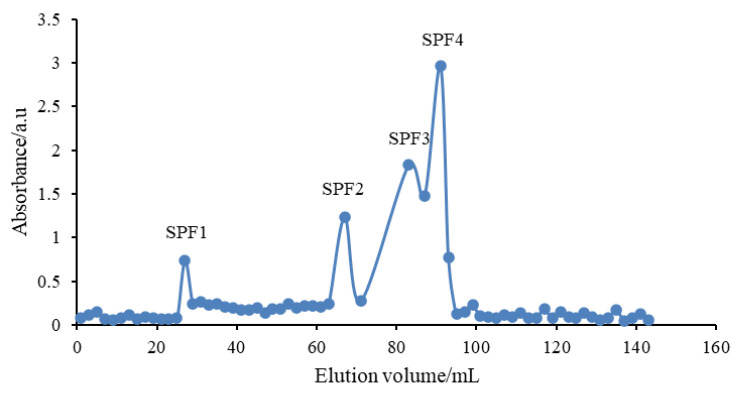
Bio Gel P10 elution curves of SPF1, SPF2, SPF3 and SPF4. Note: F: fucoidan, SPF1, SPF2, SPF3 and SPF4: fucoidan oligosaccharides.

Four oligosaccharide fractions were obtained by separation and purification using Bio Gel P10 (Figure 1) and were named SPF1, SPF2, SPF3, and SPF4. These respective yields were 3.08%, 4.6%, 17.54%, and 27.18%. These fractions were collected and stored for further use.

### 2.2. Chemical Composition Analysis of Fucoidan Oligosaccharides

#### 2.2.1. Chemical Composition

The total sugar contents of F, SPF1, SPF2, SPF3, and SPF4 were 56.80%, 65.56%, 60.66%, 65.9%, and 66.7%, respectively. The sulfate group contents were 20.34%, 13.95%, 15.30%, 15.82%, and 17.56% (Table 1). The total sugar contents of SPF1, SPF3, and SPF4 were significantly higher than those of F and SPF2. There was no significant difference in the sulfate group content between SPF2 and SPF3, while SPF4 had a higher sulfate group content of approximately 17.56%. The sulfate group content of the oligosaccharides decreased significantly after separation, which was likely due to the loss of sulfate groups during acid degradation. All polysaccharides and oligosaccharides contained mannose, rhamnose, glucose, galactose, xylose, and fucose. F and SPF2 contained a small amount of galacturonic acid. With the exception of SPF3, each component contained a small amount of glucuronic acid. The fucose contents of SPF2, SPF3, and SPF4 were higher than those of F. This indicated that the purity of the fucoidan oligosaccharides was improved after separation by the degradation column. SPF4 had the highest fucose content, at 97.09%. The high sulfate group content, total sugar content, and fucose content of SPF4 indicated that it was a more homogeneous and purer fucoidan oligosaccharide. Due to the large molecular weight of component SPF1, the HPGPC-MALLS method was chosen for its molecular weight determination. As shown in Table 1, the molecular weight range of SPF1 was 2.903 × 10^4^ to 1.365 × 10^5^ Da, with approximately 83.2% of its components having a molecular weight of 2.903 × 10^4^ Da. SPF1 was identified as a mixture.

For SPF2, SPF3, and SPF4, which have smaller molecular weights, LC-MS analysis was performed, and the results are presented in Figures S1–S3 The mass spectrometry results indicated that the SPF2, SPF3, and SPF4 components exhibited similar molecular weight distributions, almost below 1000 Da, suggesting they are low-degree of polymerization sulfonated fucoidan or fucoidan oligosaccharides. The percentage content of fucose at different degrees of polymerization and sulfation was analyzed. SPF2 was mainly composed of Fuc3S3 (54.81%), Fuc3S2 (22.13%), and Fuc2S2 (10.50%), with oligosaccharides of fucose at a polymerization degree of 3 constituting the largest proportion (81.14%). SPF3 was primarily composed of Fuc2S2 (33.96%), Fuc2S3 (18.76%), Fuc3S3 (18.65%), and Fuc3S2 (11.42%), with oligosaccharides of fucose at a polymerization degree of 2 accounting for the largest proportion (61.34%). For SPF4, it was mainly composed of Fuc2S2 (37.52%), Fuc2S3 (22.14%), and Fuc3S3 (21.53%), with oligosaccharides of fucose at a polymerization degree of 2 representing the largest proportion (62.23%). The results indicated that Bio-Gel P10 could separate the SF according to molecular weight, but complete separation of the components was not achieved.

#### 2.2.2. Structural Analysis

**Figure 2:**
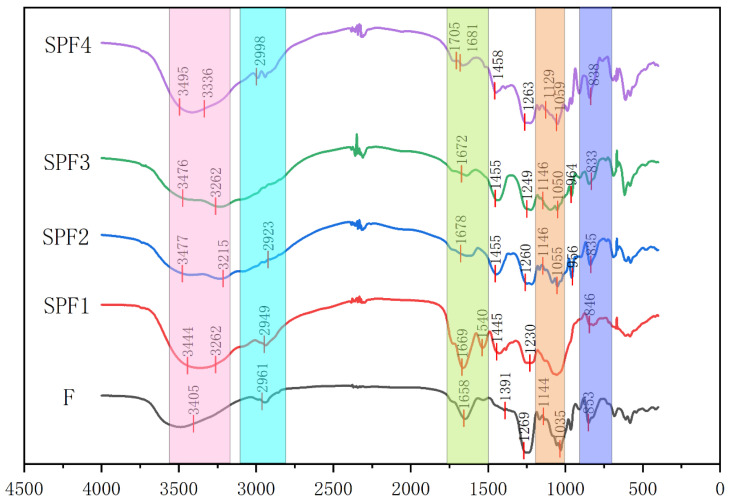
FT-IR spectra of F, SPF1, SPF2, SPF3 and SPF4. Note: F: fucoidan, SPF1, SPF2, SPF3 and SPF4: fucoidan oligosaccharides.

F, SPF1, SPF2, SPF3, and SPF4 exhibited the O-H vibrational peak around 3400 cm^−1^, the C-H vibrational peak around 2945 cm^−1^, the C=O vibrational peak near 1650 cm^−1^, the asymmetric S=O vibration around 1400 cm^−1^, the S=O vibrational peak near 1250 cm^−1^, and the stretching vibration peak around 1060 cm^−1^ corresponding to C-O-C/C-OH (Figure 2). These are characteristic peaks of carbohydrates. At 1250 cm^−1^, the absorption peaks of SPF1, SPF2, and SPF3 were lower than that of SPF4 and much lower than F. SPF4’s absorption signal was stronger than SPF3’s but weaker than F, which was consistent with the sulfate group measurement results.

The results are compared with the articles by Yuefan Song et al. and Songze K et al. [36,37]. Yuefan Song et al. reported that fucoidan was mainly composed of fucose, galactose, mannose, and glucuronic acid. The infrared (IR) spectrum of fucoidan showed a characteristic absorption band at 856–858 cm^−1^, confirming that the sulfate groups were primarily located at the axial C4 position. Songze Ke et al. found the low-molecular-weight fucoidan observed a characteristic IR absorption peak at 845.2 cm^−1^, which also indicated sulfation at the C4 position. The principal structural motif consists of α-L-fucopyranosyl residues linked via a 1→3 linkage, together with galactose-containing fucoidan. It indicated that the samples exhibit a high degree of consistency in their functional groups. The degree of polymerization (DP) of all three fractions ranged from 1 to 3. The DP of SPF3 and SPF4 was lower than that of SPF2. SPF2, SPF3 and SPF4 differed in the number of sulfate substituents. SPF2 contained a higher proportion of fucoidan trisaccharide, whereas SPF3 and SPF4 were richer in fucoidan disaccharide. All three fractions were therefore mixed-type oligosaccharides. Therefore, it could be inferred that SPF2, SPF3, and SPF4 are sulfated α-L-fucopyranoses and the sulfate groups were attached at the C4 position of fucose, specifically as sulfate groups connected via perpendicular (axial) bonds. The results still need to be further verified.

### 2.3. Symptoms of Colitis

**Figure 3:**
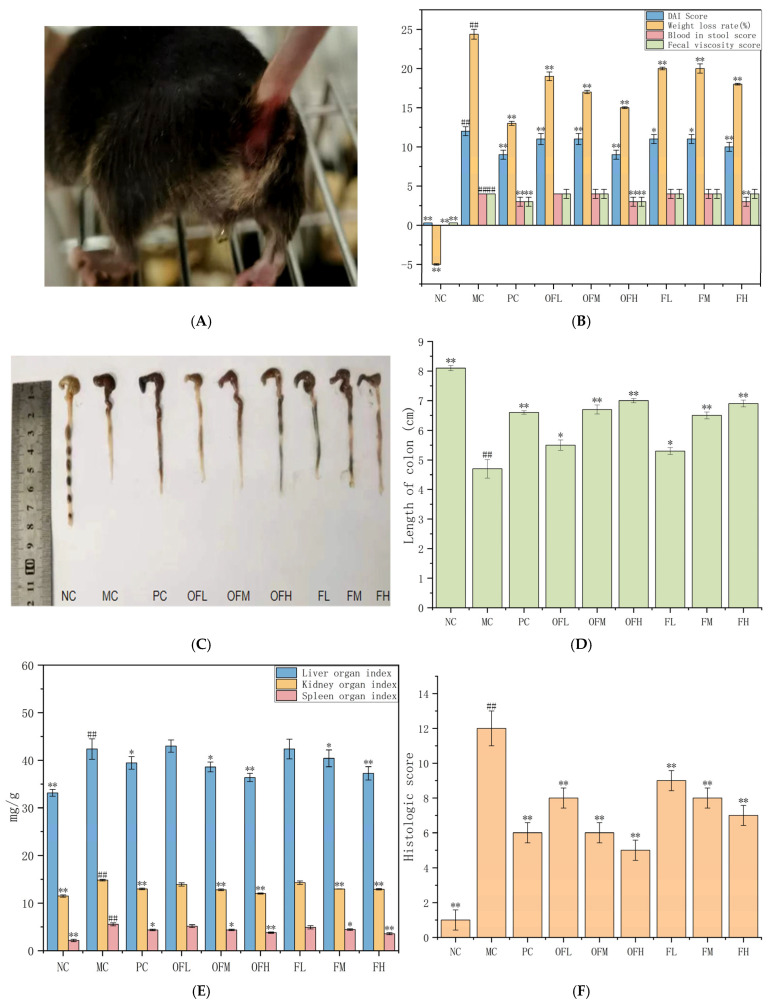

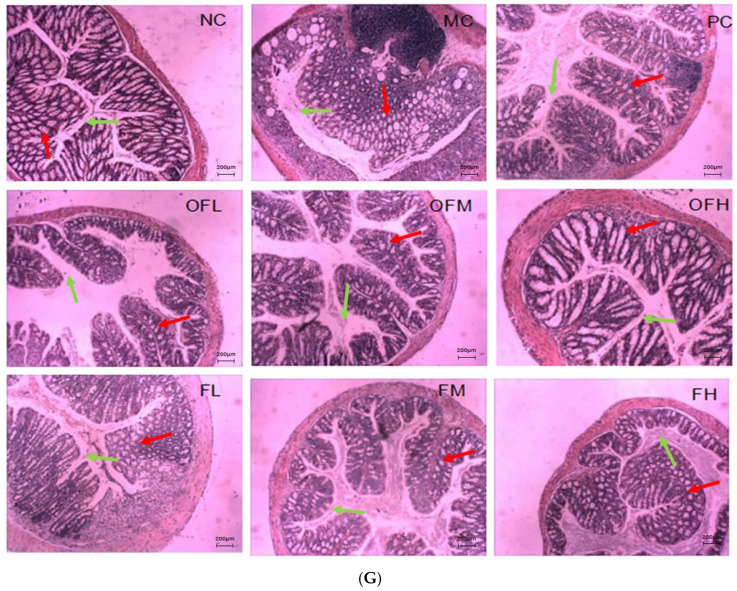
F and OF alleviate colitis symptoms in UC mice. (**A**) Anal bleeding in mice. (**B**) Colitis symptoms. (**C**) Representative images of the colon from each group. (**D**) Colon length of mice in each group. (**E**) Organ index. (**F**) Histological score. (**G**) Colon sections stained with HE. Note: Comparison between model group and control group, # *p* < 0.05, ## *p* ≤ 0.01. Comparison between the experimental groups of F and OF and the model MC group, * *p* < 0.05, ** *p* < 0.01. NC: control group, MC: model group, PC: positive control group, OFL: low-dose fucoidan oligosaccharides group, OFM: medium-dose fucoidan oligosaccharides group, OFH: high-dose fucoidan oligosaccharides group, FL: low-dose fucoidan group, FM: medium-dose fucoidan group, FH: high-dose fucoidan group.The green arrows indicate crypts, and the red arrows indicate goblet cells.

The main symptoms of colitis included weight loss, decrease in fecal consistency, diarrhea, and rectal bleeding. The Disease Activity Index (DAI) score is a comprehensive score that combines the weight loss score, stool consistency score, and fecal bleeding score. The DAI is a quantitative indicator that was used to assess the severity of colitis. Compared with the NC group, mice in the MC group exhibited symptoms such as lethargy, anorexia, diarrhea, and even bloody feces starting from the fourth day. Other groups began to show these symptoms from the fifth to sixth day. Except for the PC and OFH groups, all other groups’ mice developed anal bleeding (Figure 3A). The DAI score in the MC group was higher than in other groups. This indicated more severe inflammation. The PC, OFH, and FH groups exhibited lower DAI scores. This suggested better treatment effects. The MC group experienced the highest rate of weight loss. Other groups had lower weight loss rates than the MC group. Except for the NC group, the remaining experimental groups displayed symptoms such as loose stools and mild diarrhea from days four to five. The MC, OFL, OFM, FL, FM, and FH groups all experienced severe diarrhea, whereas only the PC and OFL groups had mild diarrhea. In summary, the OFH, FH, and PC groups showed better improvement, while the other experimental groups exhibited some alleviation of inflammatory symptoms compared to the MC group (Figure 3B). The colon length in the NC group was longer, with evenly distributed fecal particles inside the intestinal lumen, and no inflammation was observed on the intestinal surface. The colon length in the MC group was shortest, with loose stools in the lumen and damage to the intestinal wall. The colon length in the PC group was longer than in the MC group, and the intestinal wall was thicker. The protective effect of F and OF treatments on the colon was dose-dependent. Both resulted in longer colon lengths than the MC group and alleviated bowel wall edema (Figure 3C,D). These findings were consistent with previous studies by Cui et al. [38] and U et al. [39]. The results demonstrated that F and OF could reduce the severity and symptoms of DSS-induced colitis. The OFM and OFL groups were extremely significant (*p* < 0.01). OF had a more pronounced therapeutic effect on UC mice.

Organ index was an important parameter to evaluate the protective effects of substances on the immune system [40]. The livers of NC mice were red and of normal size. In the MC group, the liver became pale and slightly swollen. Kidney damage was also more severe in the MC group than in NC. Administration of F and OF mitigated the macroscopic damage to the liver and kidneys to varying degrees. Except for the OFL and FL groups, all other groups showed significant differences compared to the MC group. The spleen weight in the MC group was significantly increased. F and OF treatments effectively suppressed spleen enlargement in a dose-dependent manner. The OFH group was extremely significant (*p* < 0.01). The OFH treatment groups showed better improvement in organ indices than the F group (Figure 3E). These results suggested that F and OF could protect immune organs and reduce colitis-associated intestinal inflammation.

Histopathological examination and scoring were performed on H&E-stained colon sections. The NC group’s colon tissue appeared healthy, with intact epithelium, orderly arrangement of goblet cells and crypts, normal mucosal thickness, few lymphocytes, and normal wall thickness. The MC group showed disorganized glandular structures, mucosal detachment, and more severe tissue necrosis. The finding indicated that DSS caused significant damage to the colonic surface epithelium, distorted crypt architecture, loss of goblet cells, thickening of the outer wall, and infiltration of inflammatory cells [41]. F and OF treatments significantly restored crypt and goblet cell structures, reduced inflammatory cell infiltration, and alleviated tissue damage. The OFL, OFM and OFH groups were extremely significant (*p* < 0.01). The OF group showed better recovery of crypts, goblet cells, and a more substantial reduction in inflammation than the F group, with marked improvement in inflammatory cell infiltration (Figure 3F,G). In conclusion, the results demonstrated that fucoidan and fucoidan oligosaccharides administered via gavage effectively ameliorate DSS-induced ulcerative colitis in mice. The therapeutic effect of fucoidan oligosaccharides was stronger than that of fucoidan.

### 2.4. Oxidative Stress

**Figure 4:**
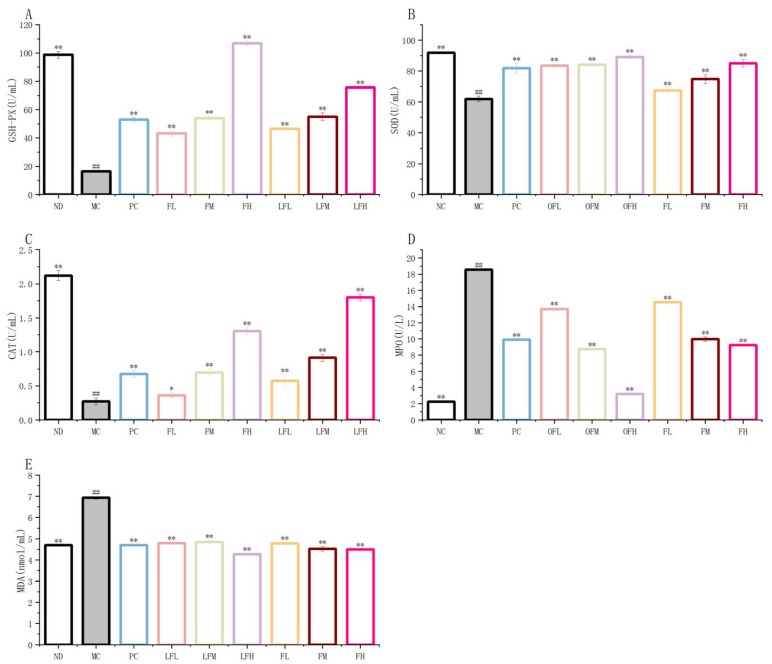
F and OF improve oxidative stress in mice. (**A**) GSH-PX. (**B**) SOD. (**C**) CAT. (**D**) MPO. (**E**) MDA. Note: Comparison between model group and control group, # *p* < 0.05, ## *p* ≤ 0.01. Comparison between the experimental groups of F and OF and the model MC group, * *p* < 0.05, ** *p* < 0.01. ND: control group, NC: control group, MC: model group, PC: positive control group, OFL: low-dose fucoidan oligosaccharides group, OFM: medium-dose fucoidan oligosaccharides group, OFH: high-dose fucoidan oligosaccharides group, FL: low-dose fucoidan group, FM: medium-dose fucoidan group, FH: high-dose fucoidan group.

Superoxide Dismutase, Glutathione Peroxidase, Catalase, Myeloperoxidase and Malondialdehyde (SOD, GSH-PX, CAT, MPO, and MDA) were oxidative stress markers associated with ulcerative colitis [42]. As shown in Figure 4, except for MPO and MDA, the levels of all markers in the MC group were significantly decreased. The result indicated that the DSS-induced inflammation was quite severe. After treatment, the levels of SOD, GSH-PX, and CAT in the F and OF experimental groups were significantly higher than those in the MC group. The levels of MPO and MDA were significantly lower. The best effects were observed in the FH and OFH groups and exhibited a dose-dependent relationship. The OF experimental groups had higher levels of SOD and CAT than the F experimental groups. The OF experimental groups showed a stronger ability to inhibit MPO and MDA activity than the F experimental groups. These findings indicated that both F and OF could significantly improve oxidative stress in mice and reduced inflammatory damage. OF had a stronger regulatory ability than F. This result suggested that low-molecular-weight fucoidan oligosaccharides had stronger antioxidant capabilities than fucoidan and might achieve better therapeutic effects in UC treatment.

### 2.5. Cytokine Levels

**Figure 5:**
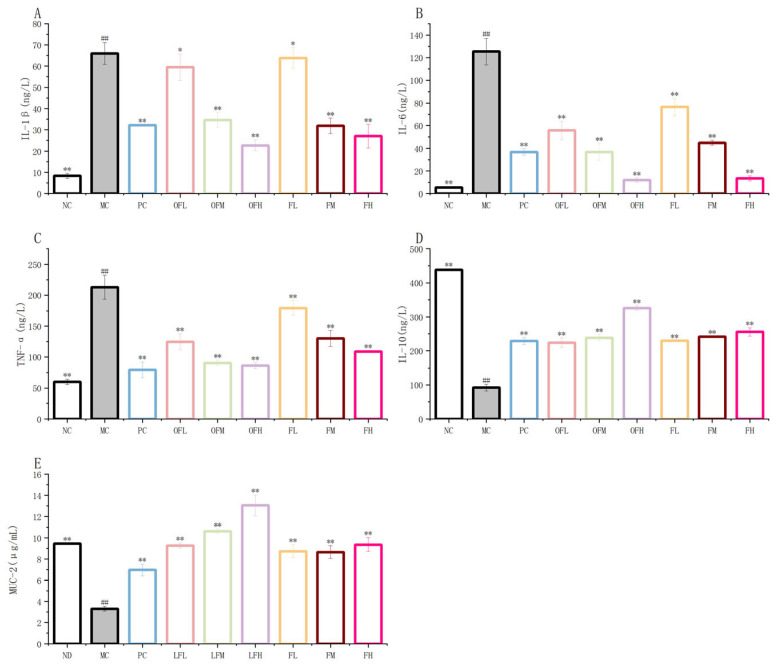
F and OF regulate cytokine and MUC-2 levels in UC mice. (**A**) IL-1β. (**B**) IL-6. (**C**) TNF-α. (**D**) IL-10. (**E**) MUC-2. Note: Comparison between model group and control group, # *p* < 0.05, ## *p* ≤ 0.01. Comparison between the experimental groups of F and OF and the model MC group, * *p* < 0.05, ** *p* < 0.01. ND: control group, NC: control group, MC: model group, PC: positive control group, OFL: low-dose fucoidan oligosaccharides group, OFM: medium-dose fucoidan oligosaccharides group, OFH: high-dose fucoidan oligosaccharides group, FL: low-dose fucoidan group, FM: medium-dose fucoidan group, FH: high-dose fucoidan group.

TNF-α, IL-1β, and IL-6 were common pro-inflammatory cytokines. IL-10 is an anti-inflammatory cytokine. The expressions of TNF-α, IL-1β and IL-6 were reduced, and the expression of IL-10 was increased. These results could alleviate colonic inflammation [43,44]. MUC-2 was the gene for the mucin core peptide and was primarily involved in encoding the intestinal mucus barrier. The expression of MUC-2 was reduced. This finding could exacerbate colonic inflammation [45]. As shown in Figure 5, gavage administration of F and OF significantly inhibited the secretion of TNF-α, IL-1β, and IL-6 and significantly promoted the production of IL-10. The F and OF experimental groups increased the MUC-2 content. Among them, the OFH group showed a stronger improvement effect than the PC and FH groups. These results indicated that F and OF could inhibit the production of pro-inflammatory cytokines and promoted the production of anti-inflammatory cytokines to alleviate DSS-induced ulcerative colitis inflammatory damage. Fucoidan oligosaccharides had a stronger effect on improving colitis than fucoidan.

### 2.6. The Expression of TLR4/MYD88/NF-κB p65/IκB-α Signaling Pathway mRNA

**Figure 6:**
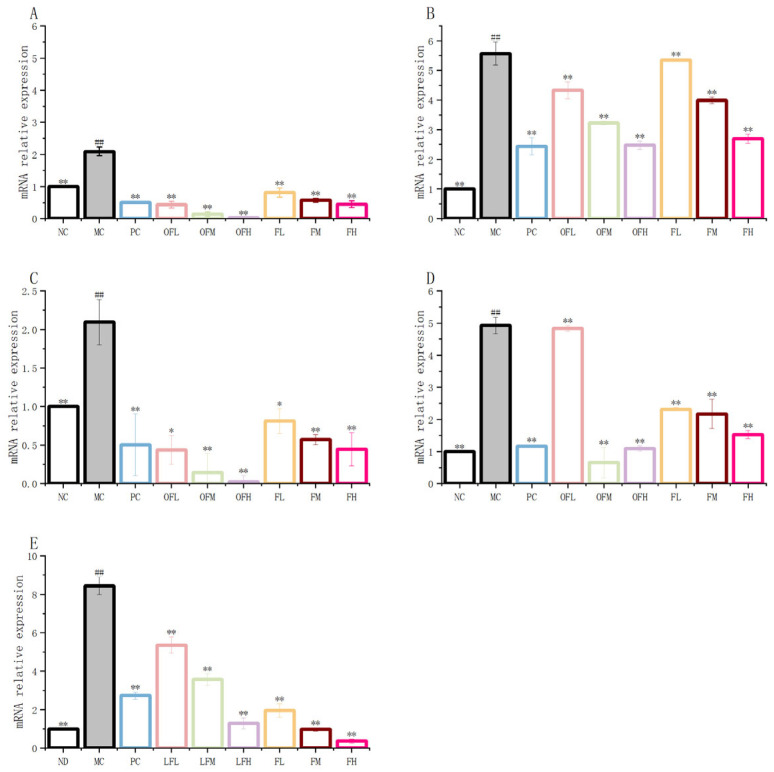
F and OF inhibit TLR4/MYD88/NF-kB/IkB-a signal pathway activation and mRNA expression level. (**A**) TLR4. (**B**) MYD88. (**C**) NF-kB. (**D**) IkB-a. (**E**) TNF-a. Note: Comparison between model group and control group, # *p* < 0.05, ## *p* ≤ 0.01. Comparison between the experimental groups of F and OF and the model MC group, * *p* < 0.05, ** *p* < 0.01. ND: control group, NC: control group, MC: model group, PC: positive control group, OFL: low-dose fucoidan oligosaccharides group, OFM: medium-dose fucoidan oligosaccharides group, OFH: high-dose fucoidan oligosaccharides group, FL: low-dose fucoidan group, FM: medium-dose fucoidan group, FH: high-dose fucoidan group.

Inhibition of the mRNA expression of various signaling pathways could improve colonic inflammation [46]. As shown in Figure 6, the RT-PCR results revealed that the relative expression levels of mRNA in various signaling pathways in the MC group were significantly increased. In the F and OF experimental groups, the relative expression levels of mRNA in these signaling pathways were markedly decreased compared to the MC group. The OFH group showed the most significant suppression of the TLR4 and NF-κBp65 signaling pathways. F and OF had similar inhibitory effects on the MYD88 signaling pathway. The OFM group exhibited the most pronounced inhibition of the IκB-α signaling pathway. The FH group had the strongest inhibitory effect on TNF-α mRNA expression. In summary, F and OF possessed strong anti-inflammatory capabilities, with the OF experimental group demonstrating a greater overall regulatory effect on signaling pathway mRNA expression than the F group. This finding indicated that low-molecular-weight fucoidan oligosaccharides had a stronger anti-inflammatory effect than fucoidan.

### 2.7. The Expression Levels of TLR4/MYD88/IκB-α/NF-κB p65 Proteins and Intestinal Barrier Proteins

**Figure 7:**
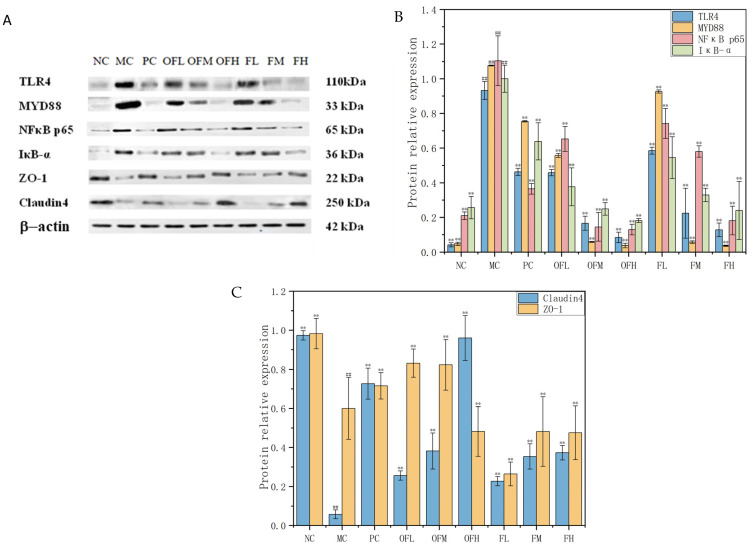
Expression levels of TLR4/MYD88/NF-κB p65/IκB-α proteins and barrier proteins. (**A**) Protein bands of each group. (**B**) Expression levels of TLR4/MYD88/NF-κB p65/IκB-α proteins. (**C**) Relative expression level of Claudin4 and ZO-1. Note: Comparison between model group and control group, # *p* < 0.05, ## *p* ≤ 0.01. Comparison between the experimental groups of F and OF and the model MC group, * *p* < 0.05, ** *p* < 0.01. NC: control group, MC: model group, PC: positive control group, OFL: low-dose fucoidan oligosaccharides group, OFM: medium-dose fucoidan oligosaccharides group, OFH: high-dose fucoidan oligosaccharides group, FL: low-dose fucoidan group, FM: medium-dose fucoidan group, FH: high-dose fucoidan group.

The production of pro-inflammatory cytokines was reduced. The expression of barrier proteins was increased. These could alleviate colonic inflammation. As shown in Figure 7, the protein expression levels of TLR4/MYD88/IκB-α/NF-κB p65 were significantly increased in the MC group. The expression levels of Claudin4 and ZO-1 proteins were significantly inhibited in the MC group. The OFH group showed a stronger ability to inhibit TLR4 protein expression. The FM group, FH group, OFM group, and OFH group showed good inhibition effects on the MYD88 signaling pathway, with very significant inhibition effects and protein expression levels close to the NC group. The most significant inhibition of NF-κB p65 protein expression was observed in the OFH group. The OFH group had the strongest effect on improving the expression levels of IκB-α protein, and almost completely preserved the function of the intestinal mucosal barrier. OF was stronger than F in restoring intestinal barrier protein levels. The F and OF experimental groups showed a dose-dependent effect. In summary, both fucoidan and fucoidan oligosaccharides could improve DSS-induced ulcerative colitis by inhibiting the expression levels of TLR4, MYD88, IκB-α, and NFκB p65 proteins and increasing the expression of intestinal barrier proteins such as Claudin4 and ZO-1.

### 2.8. Microbiota Diversity and Composition Analysis

**Figure 8:**
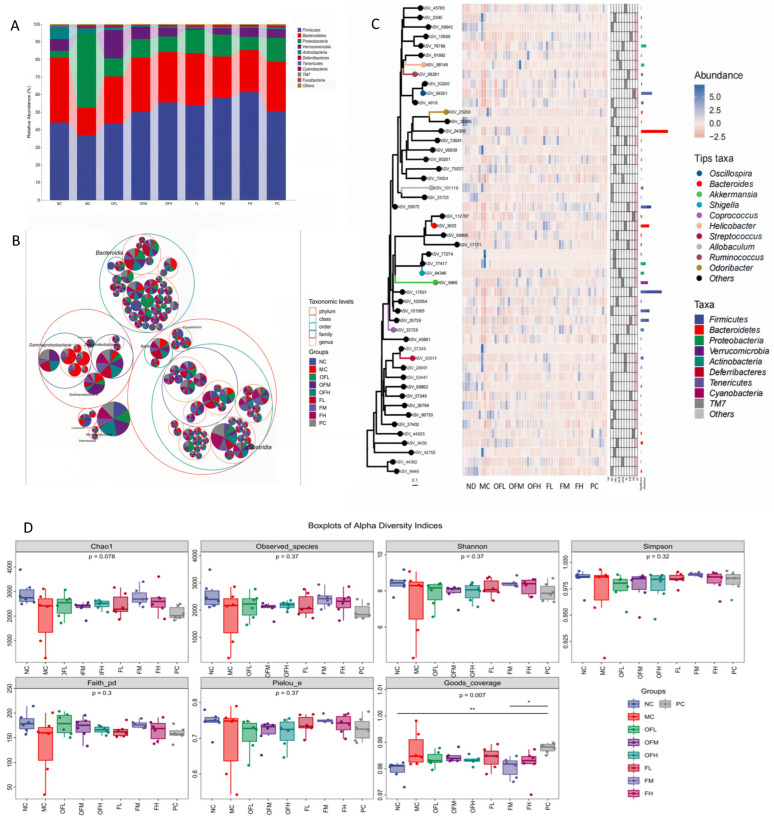

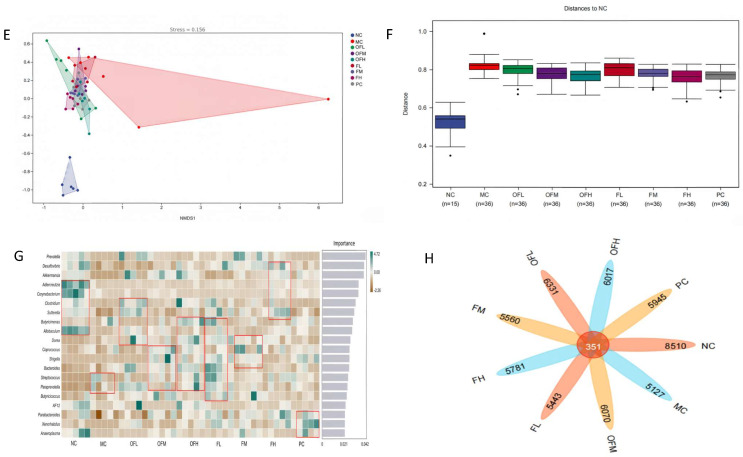
Analysis of gut microbiota composition and diversity (**A**) Species composition accumulation. (**B**) Notes on Taxonomy of Species. (**C**) Phylogenetic tree plot. (**D**) Boxplots of Alpha Diversity Indices. (**E**) NMDS analysis. (**F**) Krona circle diagram PERMANOVA analysis. (**G**) Random Forests analysis. (**H**) Petal Map Analysis of Intestinal Microbial Community. Note: The results are taken as the average of each group. NC: control group, MC: model group, PC: positive control group, OFL: low-dose fucoidan oligosaccharides group, OFM: medium-dose fucoidan oligosaccharides group, OFH: high-dose fucoidan oligosaccharides group, FL: low-dose fucoidan group, FM: medium-dose fucoidan group, FH: high-dose fucoidan group. * *p* < 0.05, ** *p* < 0.01.

Microbial diversity and abundance were characterized by 16S rRNA sequencing [47]. DNA fragments of the V4V3 region from the microbial communities were sequenced using the Illumina platform with paired-end reads. Sequences were processed to remove noise, chimeras, and were clustered. All sequences ranged from 404 to 431 base pairs in length, with varying percentages. The MC group had a higher proportion of sequences at 430 bp, while the other treatment groups had similar proportions to the NC group. Taxonomic analysis was performed at the phylum level after removing singletons. The data revealed a significant decrease in Bacteroidetes and a marked increase in Firmicutes and Proteobacteria in the MC group, with Verrucomicrobia nearly absent. These findings indicated severe colonic inflammation. Other experimental groups showed a significant decrease in Proteobacteria, a reappearance and increased proportion of Verrucomicrobia, and a reduced F/B ratio (Figure 8A). The outer circle of the taxonomic distribution from largest to smallest included Clostridium, Bacteroides, Proteobacteria, and Verrucomicrobia. Proteobacteria and Bacteroidetes were potentially harmful bacteria [48]. The relative abundance of Proteobacteria was highest in the MC group, with Proteus being the dominant genus within this phylum. Other bacteria had lower and more evenly distributed abundances across the experimental groups. These results suggested these treatments could modulate gut microbiota composition in ulcerative colitis mice (Figure 8B). The most abundant genera among the experimental groups were Bacteroides and Firmicutes. Proteus and Firmicutes were most abundant in the MC group. The difference between the MC and NC groups was the largest. Other groups showed smaller differences compared to NC (Figure 8C).

Diversity indices such as Chao1, Observed Species, Faith’s PD, and Species Good’s index indicated that DSS gavage significantly reduced microbial richness in the MC group. All treatment groups showed improvements in richness. The F group demonstrated the most notable recovery and approached the richness levels of the NC group. Shannon and Simpson indices confirmed that both the F and OF groups restored diversity of the gut microbiota in DSS-induced colitis mice, with stronger effects than the PC group. Pielou’s evenness index showed that microbial uniformity was lower in MC compared to NC, but was improved in the L and OF groups (Figure 8D). F and OF could improve colonic inflammation by regulating the composition of microbiota, increasing the proportion of beneficial bacteria, and reducing the abundance of harmful bacteria.

The NC group and MC group were positioned far apart both horizontally and vertically. The other experimental groups were located between them. This indicated that the gut microbiota of the MC group mice differs significantly from that of the NC group. The other treatment groups reduced this difference (Figure 8E). The greatest distance was observed between the MC and NC groups. The distances to other groups were shorter and varied in a dose-dependent manner (Figure 8F). The dominant genera in the NC group mainly included Corynebacterium, Akkermansia, and Allobaculum, most of which were beneficial bacteria. Allobaculum could improve metabolic syndrome and alleviate intestinal inflammation. The genus Streptococcus exacerbated colitis and other gastrointestinal diseases [49]. The abundance of microbial genera in the MC group was significantly decreased and primarily concentrated in Streptococcus. Other groups had lower abundances of various genera compared to the MC group. Clostridium was a beneficial bacterium capable of repairing intestinal mucosal proteins, which showed higher abundance in the OFL group. Butyricicoccus could control pathogenic bacterial growth and increase probiotics. The OFL and FM groups showed higher abundance of Butyricicoccus. The OFH and FL groups exhibited higher levels of Bacteroides. Parabacteroides was a beneficial genus that could improve intestinal inflammation. The FH group had more Parabacteroides. In the PC group, higher levels of Xenorhabdus and anaeroplasma were observed. Anaeroplasma was capable of degrading myo-inositol and producing short-chain fatty acids (SCFA), which are beneficial for gut health (Figure 8G). The shared operational taxonomic units (OTUs) among different groups total 351. The MC group has 5127 OTUs, which was much fewer than the 8510 OTUs in the NC group (Figure 8H). All other groups had higher OTU counts than the MC group. The OF groups exhibited the highest overall OTU number than the F groups. These findings suggested that all treatment groups could improve the gut microbiota of DSS-induced colitis mice. The microbiota of the MC group had some unique features. The NC group shared common OTUs with all other groups.

In summary, DSS gavage significantly altered the gut microbiota in the MC group mice. Treatment with F and OF reduced these differences by increasing the abundance of beneficial bacteria and decreasing the abundance of harmful bacteria. These markedly improve the diversity and composition of the gut microbiota in mice.

### 2.9. Short-Chain Fatty Acids

**Figure 9:**
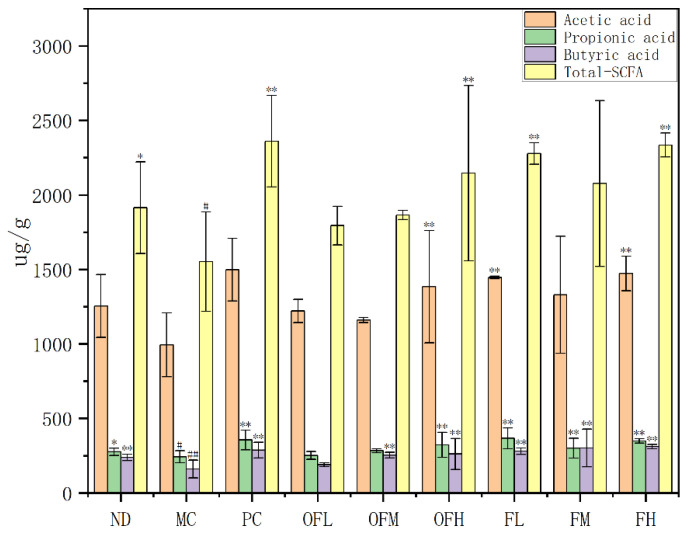
Analysis of short-chain fatty acid (SCFA) levels. Note: Comparison between model group and control group, # *p* < 0.05, ## *p* ≤ 0.01. Comparison between the experimental groups of F and OF and the model MC group, * *p* < 0.05, ** *p* < 0.01. ND: control group, MC: model group, PC: positive control group, OFL: low-dose fucoidan oligosaccharides group, OFM: medium-dose fucoidan oligosaccharides group, OFH: high-dose fucoidan oligosaccharides group, FL: low-dose fucoidan group, FM: medium-dose fucoidan group, FH: high-dose fucoidan group.

The human gut microbiota could digest dietary polysaccharides and produce functional metabolic products in the colon, such as short-chain fatty acids (SCFAs). The three main SCFAs produced by the gut microbiota were acetate, propionate, and butyrate [50]. Acetate and propionate could alleviate inflammation by inhibiting the production of harmful bacteria [51]. Butyrate maintained intestinal homeostasis through immune regulation and antioxidative stress [52]. The levels of SCFAs in the MC group significantly decreased. The overall improvement in the OF experimental groups was dose-dependent. The F experimental group showed more pronounced effects, which were generally stronger than those in the OF experimental group (Figure 9). The improvement effects of butyrate in both the OF and F groups were more significant than those of acetate and propionate. These results were similar to those reported by Li et al. [53] and Cheng et al. [54]. These findings indicated that F and OF could restore SCFA levels to reduce colitis inflammation.

## 3. Discussion and Conclusions

Fucoidan is a sulfated polysaccharide with a complex chemical structure. Fucoidan extracted from kelp has a backbone of α(1→3) l-fucose residues or alternating α(1→3) and α(1→4) linked l-fucose groups, with sulfate on C2 [55]. This was mainly composed of glucose, xylose, galactose, and mannose. Fucoidan has good biological activity, especially anti-inflammatory properties [56], which is currently the most widely used marine polysaccharide. Fucoidan can be detected at specific levels in organs, serum, and urine [57]. This might be attributable to the pharmacokinetic characteristics. Olga N. Pozharitskaya et al. reported that fucoidan exhibited a relatively long mean residence time (MRT) of 13.39 h in the kidney after oral administration [58]. This indicated that the fucoidan predominantly accumulated in the kidney. Jiaojiao Tan et al. discovered that low-molecular-weight fucoidan was absorbed more rapidly than fucoidan in mouse serum [59]. After entering the bloodstream, fucoidan could be taken up by intestinal epithelial cells in a dose-dependent manner [60]. Fucoidan oligosaccharides were absorbed more rapidly than fucoidan, which was related to the molecular weight [61]. Therefore, in this study, because of its low molecular weight and high fucose content, fucoidan oligosaccharides could enter the blood circulation more rapidly; after being absorbed by intestinal epithelial cells, they could exert effects on intestinal inflammation. Fucoidan oligosaccharides can be released from the C2 sulfation of fucoidan and have structural domains of Fuc2S, 4S, and Fuc2S6 [62]. Due to the low molecular weight and high solubility, fucoidan oligosaccharides exhibit higher biological activity than fucoidan. Fucoidan oligosaccharides may alleviate organ damage by reducing oxidative stress and improving the gut microbiota [63].

The Toll-like receptor 4 (TLR4) and nuclear factor κB (NF-κB) signaling pathways, which decline in immune function and inflammatory infiltration, are the underlying pathological mechanisms for the occurrence and exacerbation of UC. The TLR4 signaling pathway plays an important role in UC, and it is the upstream regulatory pathway of UC development and a critical signaling pathway for inflammation initiation. Myeloid differentiation primary response 88 (MyD88) is a cytoplasmic adaptor and a common linker for TLR4 pathway activation. Activated TLR4 is associated with MyD88 through its Toll/IL-1 receptor domain. Activation of MyD88 regulates downstream inflammatory pathways [64]. The NF-κB p65 signaling pathway is the downstream regulatory pathway of TLR4 and the most common signaling pathway for inflammation. Phosphorylation of IκB releases NF-κB dimers and transfers them to the nucleus, which induces the expression of inflammatory TNF-α-related genes and ulcerative colitis inflammation. Tumor necrosis factor-α (TNF-α) is derived from macrophages and monocytes in the intestine. TNF-α secretion exacerbates intestinal mucosal ulceration and intestinal inflammation. The TLR4 and MYD88 signaling pathways play a key role in the innate immune response, along with their downstream signaling pathways NF-κB p65 and IκB-α [65]. The interaction between mucosal barrier proteins and inflammatory responses plays a crucial role in the remission of UC. Intestinal barrier damage facilitates the invasion of pathogens and antigens, which can be recognized by TLR4. This result promotes the production of pro-inflammatory factors in NF-κB activation, and exacerbates colonic inflammation. Fucoidan oligosaccharides could regulate the gene levels and protein expression of the TLR4, MYD88, NF-κB P65, and IκB-α signaling pathways, which reduce the production of inflammatory factors such as TNF-α, IL-1β, and IL-6. Fucoidan oligosaccharides can also significantly increase the level of barrier proteins and reduce intestinal permeability [66]. Fucoidan oligosaccharides mitigate colonic inflammation through these mechanisms.

Gut microbial species are analyzed at the phylum level. Bacteroidetes and Firmicutes are the most dominant phyla in the gut, with Bacteroidetes being a beneficial bacterial group. Bacteroidetes can secrete hydrolases to degrade polysaccharides into smaller carbohydrate molecules. Its high polysaccharide utilization ability can provide suitable glycans for other organisms. The increase in Firmicutes leads to excessive energy storage and intestinal endocrine disorders. The Firmicutes/Bacteroidetes (F/B) ratio can be used as a biomarker associated with intestinal dysfunction to identify pathological conditions of intestinal microorganisms. A low F/B ratio is associated with a balanced immune system and is generally considered beneficial to health [67]. Verrucomicrobia is a beneficial bacterium that can degrade mucosal mucins proteins in the intestinal wall and has good colon protection ability. A typical feature of UC is a large number of Proteobacteria, which increases colonic susceptibility. These lead to defects in the innate and adaptive immune systems of DSS-induced mice. Fucoidan oligosaccharides restore gut microbiota diversity and improve colonic inflammation by increasing the proportion of beneficial bacteria such as Bacteroidetes and Verrucomicrobia, and reducing the abundance of harmful bacteria such as Firmicutes, Proteobacteria, and other protein bacteria.

Although this study confirmed the anti-inflammatory activity of fucoidan and fucoidan oligosaccharides and identified partial mechanisms by examining the expression of key genes and proteins in the TLR4 and NF-κB pathway, further investigation is needed into the structure of fucoidan oligosaccharides and the mechanisms by which they exert their anti-inflammatory activity and improve intestinal flora activity. Moreover, the animal study used an acute DSS-induced colitis model, which only reflected the early stage of inflammation and lacked validation in chronic or recurrent inflammatory settings. While the mouse gut microbiota contained microbial taxa that were functionally equivalent to those in the human gut microbiota, both can produce short-chain fatty acids that help alleviate intestinal inflammation [68]. However, because of the numerous interspecies differences such as microbiota, immune systems, and other aspects, many differences still exist. Consequently, this study could only demonstrate that fucoidan oligosaccharides exerted a certain ameliorative effect on intestinal inflammation in mice. The impact on the human gut microbiota remained to be investigated. Future research should expand structural analyses, incorporate additional disease models, and conduct more systematic investigations to enhance the reliability and translational potential of the conclusions.

In conclusion, the results showed that fucoidan and fucoidan oligosaccharides significantly alleviated DSS-induced colonic tissue damage, reduced inflammatory cytokines (TNF-α, IL-1β, IL-6), and improved oxidative stress markers. Mechanistic studies indicated that both agents mainly act by inhibiting activation of the TLR4 and NF-κB signaling pathways, thereby ameliorating intestinal inflammation. This study systematically elucidates the protective role of fucoidan oligosaccharides in the DSS-induced ulcerative colitis model and provides theoretical and experimental support for the further development of low-molecular-weight algal sugars as functional anti-inflammatory foods.

## 4. Materials and Methods

### 4.1. Materials and Reagents

Fucoidan from *Kjellmaniella crassifolia*, named “F”, was cultured in the Lvshun Sea area of Dalian, China. The algae were harvested in June 2022 and identified as *Kjellmaniella crassifolia* by Professor Zeyu Zhang from the Key Laboratory of Mariculture & Stock Enhancement in the North China’s Sea, Ministry of Agriculture and Rural Affairs. The harvested algae were sun-dried to remove most of their moisture for storage. After sun-drying, the dried materials were stored in a cool, dry environment for later use (National R&D Branch Center for Seaweed Processing of Dalian Ocean University, Dalian, China). Bio-Gel P10 (American Bio-Rad Corporation, Hercules, CA, USA). Dextran Sulfate Sodium (DSS, M.W. 40,000) (Shanghai Bi-De Pharmaceutical Technology Co., Ltd., Shanghai, China). Assay Kits for Catalase (CAT), Myeloperoxidase (MPO), Superoxide Dismutase (SOD), Malondialdehyde (MDA), and Glutathione Peroxidase (GSH-PX), ELISA Kits for Mouse Interleukin-6 (IL-6), Mouse Interleukin-10 (IL-10), Mouse Tumor Necrosis Factor-α (TNF-α), and Mouse Mucin-2 (MUC-2) (Nanjing Jiancheng Bioengineering Institute, Nanjijng, China). Universal RNA Extraction Kit, SYBR Green Pro Taq HS Premixed qPCR Kit, Evo M-MLV Reverse Transcription PreMix (Hunan Accurate Bio-Medicine Technology Co., Ltd., Hunan, China). RIPA Lysis Buffer (Beyotime Biotechnology, Shanghai, China). Pierce™ Rapid Gold BCA Protein Assay Kit (Thermo Fisher Scientific, Shanghai, China).

### 4.2. Preparation of Fucoidan Oligosaccharides

Fucoidan was added to the 0.2 mol/L H_2_SO_4_, and the pH was adjusted to 3. The mixture was then transferred to a reactor and heated at 110 °C for 3 h to carry out acid hydrolysis. After the reaction was completed, the mixture was cooled to room temperature, the pH was readjusted to 7, and the sample was centrifuged to collect the supernatant [69]. F was precipitated with an equal volume of ethanol (1:1, *v*/*v*), which was allowed to stand overnight and centrifuged. The supernatant was rotary evaporated and followed by lyophilization. The resulting product was named SF. SF was purified using a Bio-Gel P10 (1.6 cm × 100 cm) gel filtration column with 0.5 mol/L NH_4_HCO_3_ as the mobile phase at a flow rate of 0.15 mL/min. The eluate was then rotary evaporated and lyophilized to yield four fractions, which were named SPF1, SPF2, SPF3, and SPF4.

### 4.3. Chemical Composition Analysis of Fucoidan Oligosaccharides

The total carbohydrate content was determined using the phenol sulfuric acid method [70]. The sulfate groups were measured by the barium sulfate turbidimetric method [71]. The monosaccharide composition of F, SPF1, SPF2, SPF3, and SPF4 was analyzed by high-performance liquid chromatography (HPLC) [72]. The molecular weights of SPF2, SPF3, and SPF4 were determined by liquid chromatography–mass spectrometry (LC-MS). Due to the larger molecular weight of SPF1, the molecular weight of SPF1 was measured using high-performance gel permeation chromatography (HPGPC) combined with multi-angle laser light scattering (MALLS) [73]. The infrared spectrum was obtained using the potassium bromide (KBr) pellet method [74].

### 4.4. Animal Experiment and Handling

Ninety male SPF-grade C57BL/6 mice (weighing 20–25 g, 6 weeks old) were purchased from Liaoning Changsheng Biotechnology Co., Ltd. (License No. SCXK (Liao) 2020-0001). The male C57BL/6 mice were randomly divided into nine groups (*n* = 10 per group): control group (NC), model group (MC), low-dose fucoidan oligosaccharides group (OFL), medium-dose fucoidan oligosaccharides group (OFM), high-dose fucoidan oligosaccharides group (OFH), low-dose fucoidan group (FL), medium-dose fucoidan group (FM), high-dose fucoidan group (FH), and positive control group (PC). The mice were housed at a constant temperature of 25 °C under a 12 h light/dark cycle and were given free access to food and water for one week prior to the experiment. All animal housing and experimental procedures were conducted in accordance with the “Regulations for the Administration of Laboratory Animals in China” and were approved by the Animal Ethics Committee of Dalian Ocean University (Approval No. DLOU2023011).

### 4.5. Establishment of UC Mice Model

Following the method described by Wang [75], 3% DSS solution was used to induce a mice model of ulcerative colitis. After a one-week acclimation period, the mice were randomly divided into the following groups: control group (NC), model group (MC), low-dose fucoidan oligosaccharides group (OFL, 50 mg/kg/d), medium-dose fucoidan oligosaccharides group (OFM, 100 mg/kg/d), high-dose fucoidan oligosaccharides group (OFH, 200 mg/kg/d), low-dose fucoidan group (FL, 50 mg/kg/d), medium-dose fucoidan group (FM, 100 mg/kg/d), high-dose fucoidan group (FH, 200 mg/kg/d), and positive control group (PC, 200 mg/kg/d). Except for the NC group, all other groups were freely provided with 3% DSS solution daily. Gavage administration was performed at the same time each day. The PC group received sulfasalazine (Sulfasalazine, SASP, 200 mg/kg/d). The NC and MC groups received physiological saline (200 mg/kg/d). The experimental groups received corresponding doses of fucoidan oligosaccharide (OF) and fucoidan (F), with OF prepared from SPF4 as described in Section 4.3. The samples were dissolved in distilled water to prepare a solution of a certain concentration. The mice were weighed daily, and were administered orally at a standard dose of 0.1 mL per 10 g of body weight. The experiment lasted for seven days, with body weight recorded daily. After the final gavage, the mice were fasted for 24 h, weighed, and then euthanized via cervical dislocation after blood collection from the eyeballs. The fresh liver, spleen, and kidney were collected to determine organ indices.

### 4.6. Disease Activity Index (DAI) Assessment

The Disease Activity Index (DAI) is a quantitative measure used to assess the severity of colitis damage. The DAI was measured daily in the animal experiment, which was based on body weight loss, stool consistency, and the presence of blood in the stool [76]. The calculation method is shown in Table 2.

### 4.7. Colon Histopathological Analysis

The distal colon tissue (1–2 cm) was collected and immersed in 4% paraformaldehyde solution. The processed colon tissue was embedded in liquid paraffin. The paraffin block was sectioned into 5–10 μm thick tissue slices using a microtome. The sections were stained with hematoxylin and eosin (H&E) dyes. The morphology and structure of the colon tissue were observed under a light microscope for histological evaluation. The inflammation, extent of mucosal damage, tissue repair, crypt damage, and percentage of colon damage were assessed for each colon sample [77]. The scoring method is shown in Table 3.

### 4.8. Measurement of Oxidative Stress

Mice whole blood was collected from the mice via retro-orbital bleeding. The blood was centrifuged at 4 °C for 20 min to obtain the serum. The levels of CAT, SOD, GSH-Px, MDA, and MPO in the mice serum were measured according to the instructions provided in the ELISA kit.

### 4.9. Measurement of Cytokine Levels

An amount of 200 mg of the colon tissue was homogenized with PBS (phosphate-buffered saline, 1:9, *w*/*v*), which was centrifuged for 20 min (4000 rpm, 4 °C) and the supernatant was collected. The colon tissue homogenate was measured for the levels of pro-inflammatory cytokines TNF-α, IL-1β, IL-6, and the anti-inflammatory cytokine IL-10, as well as mucin MUC-2.

### 4.10. Determination of TLR4/MYD88/NF-κB/IκB-α/TNF-α mRNA Expression

RNA was extracted from 20 mg of colon tissue, which was added to lysis buffer. The RNA purity was determined using the microvolume spectrophotometer. The extracted RNA was reverse transcribed using the Evo M-mLV reverse transcription premix. The primer designs for the TLR4, MYD88, NF-κB p65, IκB-α and TNF-α genes in C57BL/6 mice are listed in Table 4. The relative expression level of the target genes normalized to the reference gene was determined using the 2^−ΔΔQq^ method.

### 4.11. Western Blot Analysis

Cells and tissues were lysed in RIPA buffer. The clarified supernatant was collected to obtain adherent cell total protein lysate, suspended cell total protein lysate, tissue total protein lysate, and nuclear protein lysate. For each sample, total protein solution was mixed with loading buffer, vortexed, and heated at 98 °C for 10 min. According to the molecular weight of the proteins, 10% separating gel and 5% stacking gel were prepared. After adding TEMED, the gels were cast and allowed to polymerize. During electrophoresis, the stacking gel was run at 80 V and the separating gel at 130 V. When the protein bands reached the bottom of the glass plate, the electrophoresis was stopped, and the proteins were transferred to a membrane. The membrane was blocked and incubated on a shaker, followed by incubation with primary antibodies diluted appropriately, then with secondary antibodies. The bands were exposed, developed, and analyzed. Finally, the target band densities were quantified using ImageJ 1.53 software for system analysis [78].

### 4.12. Gut Microbiota Analysis

Nucleic acids were extracted using the OMEGA Soil DNA Kit (D5625-01) (Proliant Health Biologicals (Tianjin, China) Biotechnology Co., Ltd., Tianjin, China). Agarose gel electrophoresis was performed to assess molecular size. The UV spectrophotometer (Shanghai Bajiu Industrial Co., Ltd., Shanghai, China) was used to quantify the DNA. The V3 and V4 regions of the 16S rRNA gene were amplified by PCR for sequencing [79]. The PCR products were quantified by the Quant-iT PicoGreen dsDNA Assay Kit on a Microplate reader (BioTek, FLx800, Winooski, VT, USA), and pooled according to the required data amount for each sample. Library preparation was performed by the TruSeq Nano DNA LT Library Prep Kit (Illumina, Inc., San Diego, CA, USA). The DADA2 method in QIIME2 software (Northern Arizona University, Flagstaff, AZ, USA) was used for sequence denoising. The length distribution of high-quality sequences contained in all samples was statistically analyzed for subsequent species composition analysis, abundance analysis, and species difference analysis.

### 4.13. Short-Chain Fatty Acid (SCFA) Measurement

The content of short-chain fatty acids in the proximal colon and cecum was analyzed by GC-MS and a TRACE1310-ISQLT system. Separation was performed by the Agilent DB-FFAP capillary column (30 m × 250 μm × 0.25 μm) in a gas chromatography system. The temperature program was as follows: an initial temperature of 90 °C, the temperature was increased to 160 °C at a rate of 10 °C/min, which followed increasing to 240 °C at a rate of 40 °C/min and held for 5 min. Helium was used as the carrier gas at a flow rate of 1.0 mL/min. Mass spectrometry conditions: mass spectrometric analysis was performed by the mass spectrometer with an injection port temperature of 250 °C, an ion source temperature of 230 °C, a transfer line temperature of 250 °C, and a quadrupole temperature of 150 °C. An electron impact ionization (EI) source was used with an electron energy of 70 eV. The analytes were detected using SCAN/SIM mode.

### 4.14. Statistical Analysis

Each experiment had three replicates, and the results were expressed as mean ± standard deviation. Data were analyzed using SPSS 17.0 by one-way ANOVA. A *p*-value less than 0.05 (*p* < 0.05) was considered statistically significant. A *p*-value less than 0.01 (*p* < 0.01) was considered highly statistically significant.

## Figures and Tables

**Table 1:** Chemical constituents of F, SPF1, SPF2, SPF3 and SPF4.

Sample	F	SPF1	SPF2	SPF3	SPF4
Total sugar (%)	56.80 ± 0.78 ^c^	65.56 ± 0.68 ^a^	60.66 ± 0.99 ^b^	65.92 ± 1.37 ^a^	66.70 ± 1.13 ^a^
Sulfate group (%)	20.34 ± 1.25 ^a^	13.95 ± 0.55 ^c^	16.30 ± 0.60 ^b^	15.82 ± 0.81 ^b^	17.56 ± 0.44 ^b^
Range of molecular weight (Da)	4.34 × 10^5^	2.9 × 10^4^~1.36 × 10^5^	182~1012	161~939	161~939
Composition of monosaccharides (%)					
Fucose	92.59	23.53	96.99	96.15	97.09
Xylose	0.85	11.13	0.87	1.32	0.97
Rhamnose	0.79	0.29	0.04	0.18	0.12
Mannose	0.37	42.69	0.27	0.37	0.25
Galactose	2.43	16.65	0.59	1.35	0.70
Glucuronic acid	0.23	0.99	0.10	-	0.19
Galacturonic acid	0.22	-	0.09	-	-
Glucose	2.46	4.74	0.18	0.35	1.22

Note: F: fucoidan, SPF1, SPF2, SPF3 and SPF4: fucoidan oligosaccharides. Different letters in the same row represent significant differences between different samples at the same period (*p* < 0.05).

**Table 2:** Disease Activity Index (DAI).

Score	Weight Loss/%	Stool Consistency	Rectal Bleeding
0	0	Normal Stool	No Blood in Stool
1	1–5	Soft Stool	No Blood in Stool
2	5–10	Soft Stool	Occult Blood in Stool
3	10–20	Watery/Loose Stool	Blood in Stool
4	>20	Watery/Loose Stool	Anal Bleeding

DAI Score = Weight Loss Score + Stool Consistency Score + Bleeding Score.

**Table 3:** Histological evaluation of colon.

Feature Grading	Score	Description
Inflammation	0	None
	1	Mild
	2	Moderate
	3	Severe
Degree of Mucosal Damage	0	None
	1	Mild Mucosal Damage
	2	Damage to Mucosa and Submucosa
	3	Damage to Mucosal Wall
Tissue Repair	4	No Tissue Repair
	3	Incomplete Epithelium
	2	Regeneration with Crypt Depletion
	1	Almost Complete Regeneration
	0	Completely Regenerated or Returned to Normal Tissue
Crypt Damage	0	None
	1	1/3 Damaged
	2	2/3 Damaged
	3	Only Epithelium Intact
	4	Entire Crypt and Epithelium Lost
Percentage of Colon Damage	1	1–25%
	2	26–50%
	3	51–75%
	4	76–100%

**Table 4:** Primer design.

Gene	Sequence Content
TLR4	F: TCCTGTGGACAAGGTCAGCAAC
	R: TTACACTCAGACTCGGCACTTAGCA
MYD88	F:TACAGGTGGCCAGAGTGGAA
	R:GCAGTAGCAGATAAAGGCATCGAA
P65 NF-kB	F:ATTGCTGTGCCTACCCGAAAC
	R:TTTGAGATCTGCCCTGATGGTAA
IkB-a	F:TTGATTGAACCACCATAGACCTA
	R:TTACAAGAAGGCGACACAGAC
TNF-a	F:CCCTTTACTCTGACCCCTTTATTGT
	R:TGTCCCAGCATCTTGTGTTTCT
b-actin	F:CATCCGTAAAGACCTCTATGCCAAC
	R:ATGGAGCCACCGATCCACA

## Data Availability

The original contributions presented in this study are included in the article; further inquiries can be directed to the corresponding author.

## Supplementary Materials

The following supporting information can be downloaded at: https://www.mdpi.com/article/10.3390/md24050186/s1, Figure S1: Mass spectrum of each component of SPF2; Figure S2: Mass spectrum of each component of SPF3; Figure S3: Mass spectrum of each component of SPF4.
