# Supplementary material for: Fucoidan Oligosaccharides from Kjellmaniella crassifolia Ameliorate Ulcerative Colitis by Regulating the TLR4 and NF-κB Signaling Pathway and Modulating Gut Microbiota
Source: Mar Drugs. 2026 May 21;24(5):186. doi: 10.3390/md24050186 (PMC13209098; doi:10.3390/md24050186)
Supplement: Supplementary file 1 [file marinedrugs-24-00186-s001.zip › marinedrugs-4273841-supplementary.pdf]

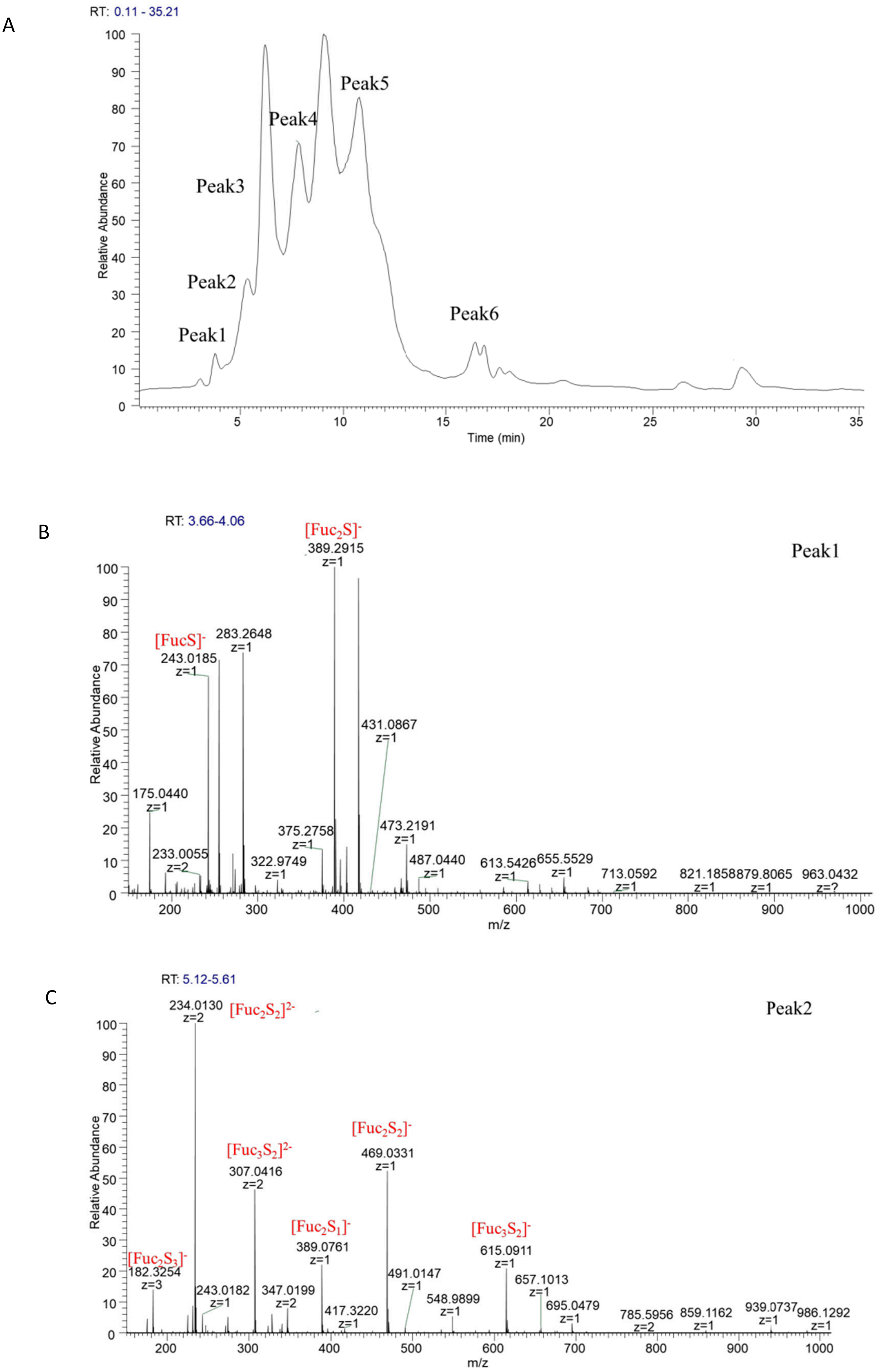

A
RT: 0.11 - 35.21
Relative Abundance
Time (min)
Peak1
Peak2
Peak3
Peak4
Peak5
Peak6
B
RT: 3.66-4.06
Peak1
[FucS]-
[Fuc2S]-
389.2915 z=1
283.2648 z=1
243.0185 z=1
431.0867 z=1
175.0440 z=1
473.2191 z=1
375.2758 z=1
233.0055 z=2
487.0440 z=1
322.9749 z=1
613.5426 z=1
655.5529 z=1
713.0592 z=1
821.1858 z=1
879.8065 z=1
963.0432 z=?
m/z
C
RT: 5.12-5.61
Peak2
234.0130 z=2
[Fuc2S2]2-
[Fuc2S2]-
469.0331 z=1
[Fuc3S2]2-
307.0416 z=2
[Fuc2S1]-
389.0761 z=1
[Fuc3S2]-
615.0911 z=1
[Fuc2S3]-
182.3254 z=3
491.0147 z=1
657.1013 z=1
243.0182 z=1
347.0199 z=2
548.9899 z=1
695.0479 z=1
417.3220 z=1
785.5956 z=2
859.1162 z=1
939.0737 z=1
986.1292 z=1
m/z

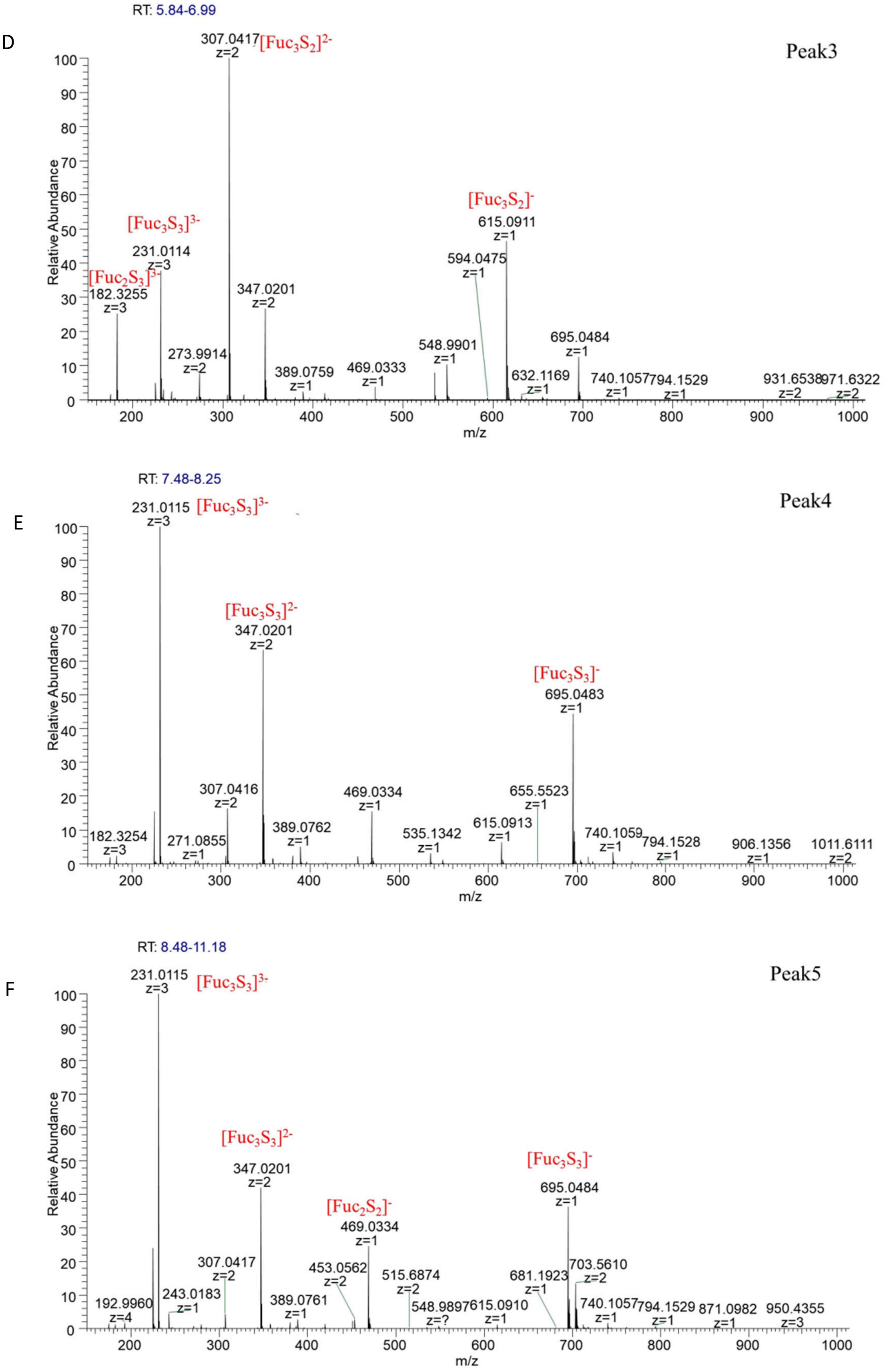
D
RT: 5.84-6.99
Peak3
307.0417 [Fuc3S2]2-
z=2
[Fuc3S3]3-
231.0114
z=3
[Fuc2S3]3-
182.3255
z=3
347.0201
z=2
273.9914
z=2
389.0759
z=1
469.0333
z=1
548.9901
z=1
594.0475
z=1
[Fuc3S2]-
615.0911
z=1
632.1169
z=1
695.0484
z=1
740.1057
z=1
794.1529
z=1
931.6538
z=2
971.6322
z=2
Relative Abundance
m/z
E
RT: 7.48-8.25
Peak4
231.0115 [Fuc3S3]3-
z=3
[Fuc3S3]2-
347.0201
z=2
[Fuc3S3]-
695.0483
z=1
182.3254
z=3
271.0855
z=1
307.0416
z=2
389.0762
z=1
469.0334
z=1
535.1342
z=1
615.0913
z=1
655.5523
z=1
740.1059
z=1
794.1528
z=1
906.1356
z=1
1011.6111
z=2
Relative Abundance
m/z
F
RT: 8.48-11.18
Peak5
231.0115 [Fuc3S3]3-
z=3
[Fuc3S3]2-
347.0201
z=2
[Fuc2S2]-
469.0334
z=1
[Fuc3S3]-
695.0484
z=1
192.9960
z=4
243.0183
z=1
307.0417
z=2
389.0761
z=1
453.0562
z=2
515.6874
z=2
548.9897
z=?
615.0910
z=1
681.1923
z=1
703.5610
z=2
740.1057
z=1
794.1529
z=1
871.0982
z=1
950.4355
z=3
Relative Abundance
m/z

G

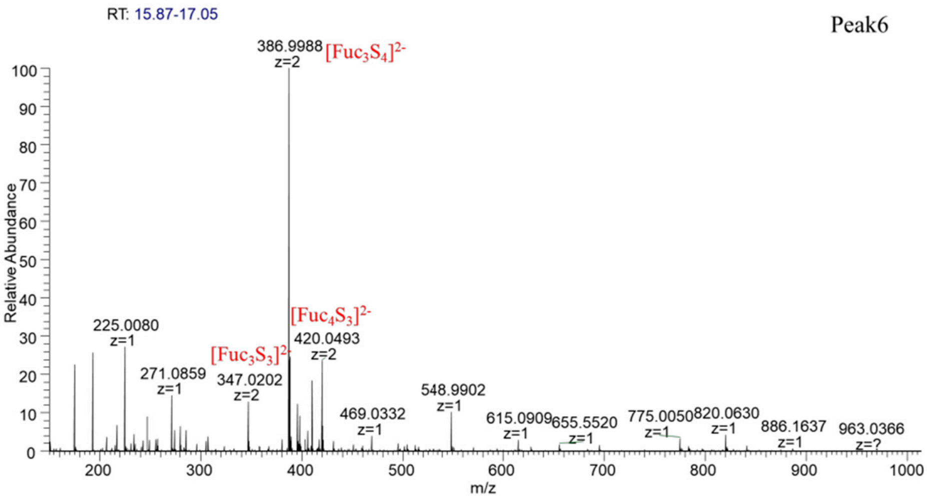


Figure S1. Mass spectrum of each component of SPF2, A: TIC diagram of SPF2 liquid quality, B: SPF2-1, C: SPF2-2, D: SPF2-3, E: SPF2-4, F: SPF2-5, G: SPF2-6.

A

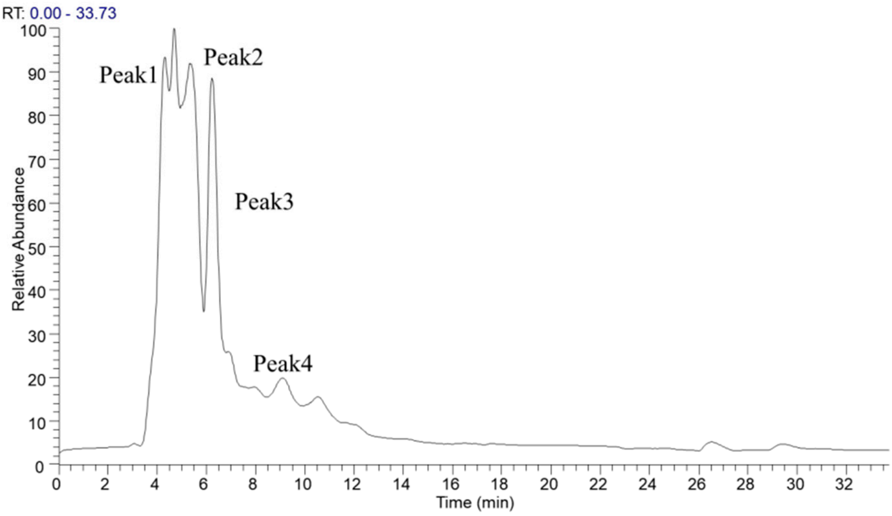


B

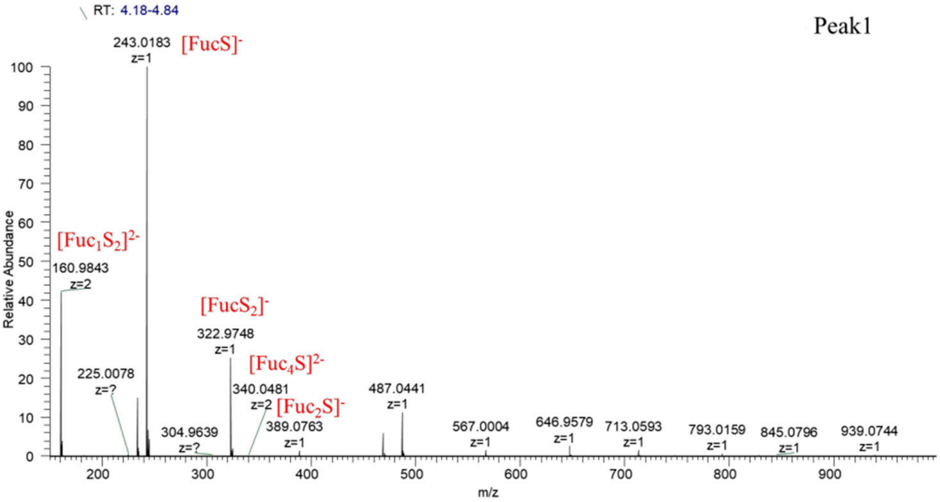

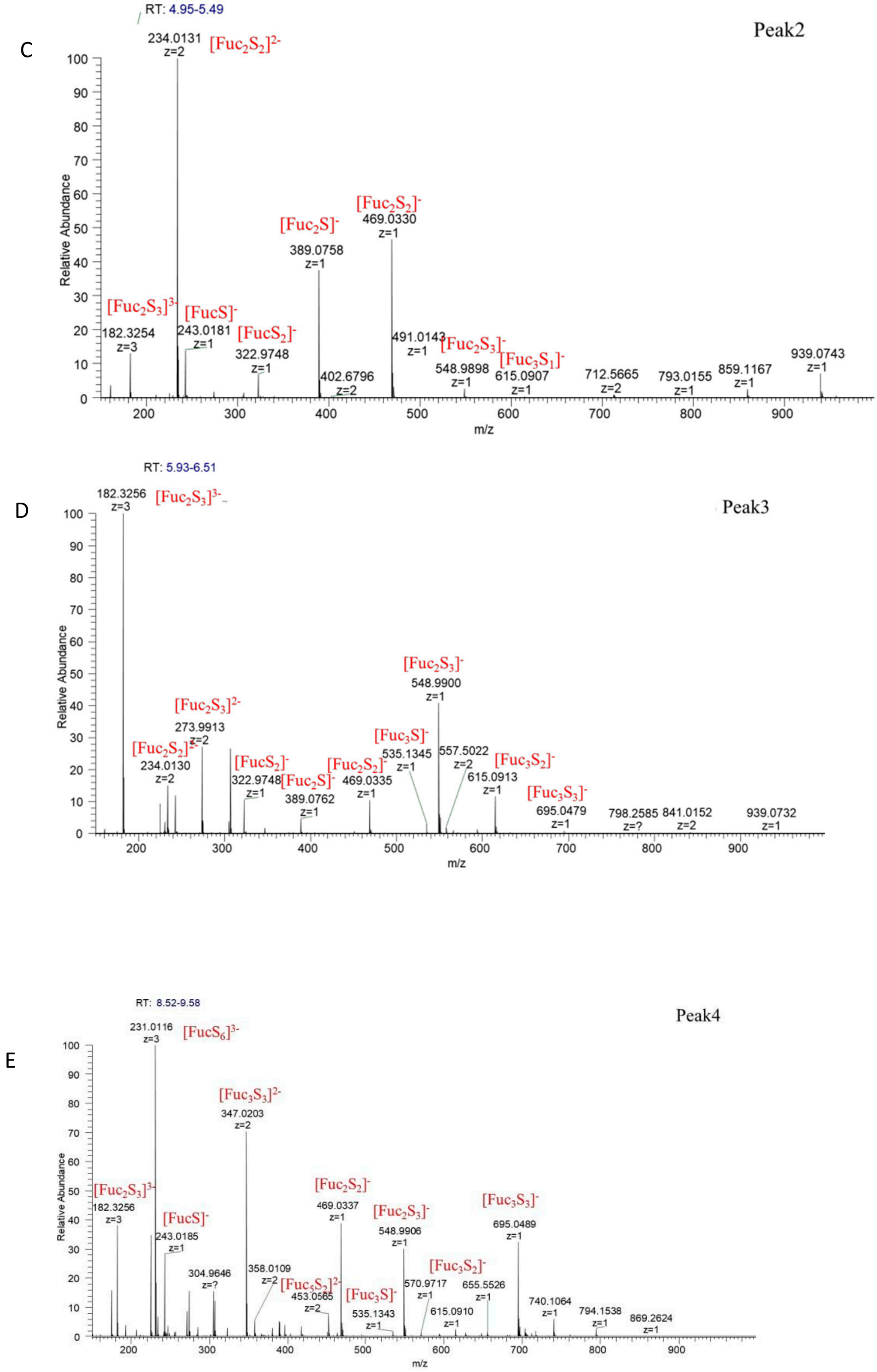


Figure S2. Mass spectrum of each component of SPF3, A: TIC diagram of SPF3 liquid quality, B: SPF3-1, C: SPF3-2, D: SPF3-3, E: SPF3-4.

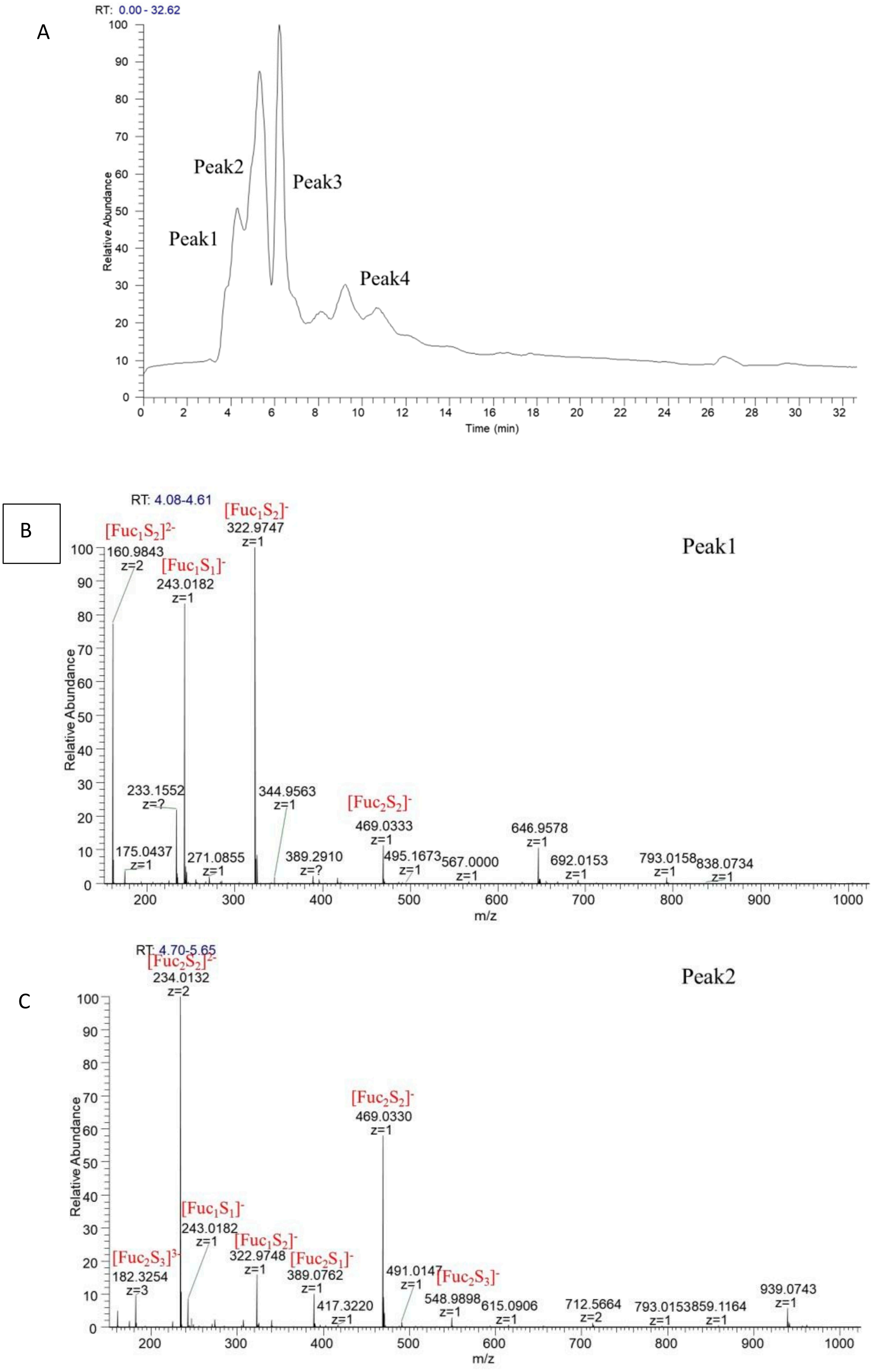
A
RT: 0.00 - 32.62
Peak1
Peak2
Peak3
Peak4
Relative Abundance
Time (min)
B
RT: 4.08-4.61
Peak1
[Fuc1S2]2-
160.9843
z=2
[Fuc1S1]-
243.0182
z=1
[Fuc1S2]-
322.9747
z=1
233.1552
z=?
344.9563
z=1
[Fuc2S2]-
469.0333
z=1
646.9578
z=1
175.0437
z=1
271.0855
z=1
389.2910
z=?
495.1673
z=1
567.0000
z=1
692.0153
z=1
793.0158
z=1
838.0734
z=1
Relative Abundance
m/z
C
RT: 4.70-5.65
Peak2
[Fuc2S2]2-
234.0132
z=2
[Fuc2S2]-
469.0330
z=1
[Fuc1S1]-
243.0182
z=1
[Fuc1S2]-
322.9748
z=1
[Fuc2S1]-
389.0762
z=1
[Fuc2S3]3-
182.3254
z=3
491.0147
z=1
[Fuc2S3]-
548.9898
z=1
417.3220
z=1
615.0906
z=1
712.5664
z=2
793.0153
z=1
859.1164
z=1
939.0743
z=1
Relative Abundance
m/z

D

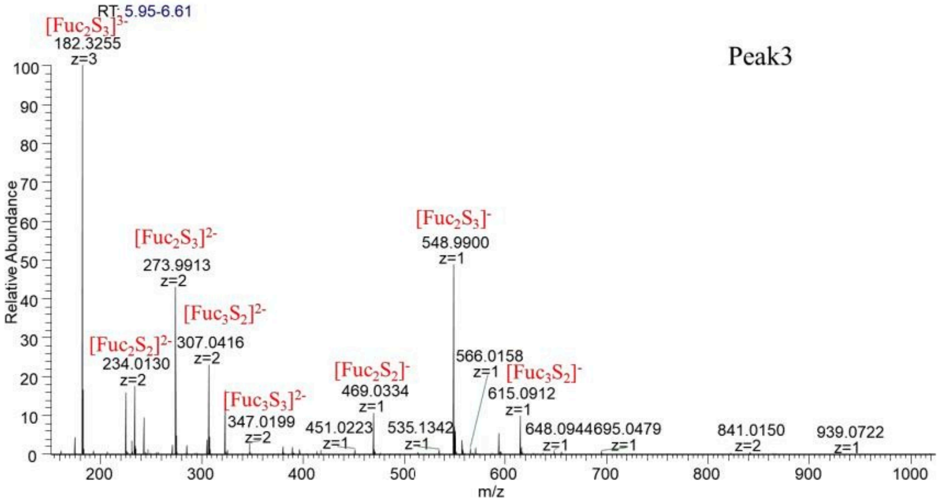


E

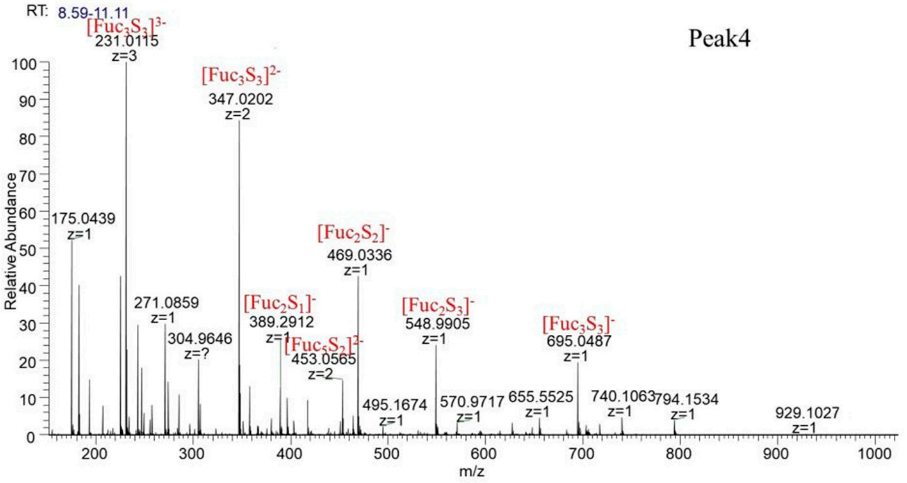


Figure S3 Mass spectrum of each component of SPF4, A: TIC diagram of SPF4 liquid quality, B: SPF4-1, C: SPF4-2, D: SPF4-3, E: SPF4-4.
